# Inter-brain desynchronization in social interaction: a consequence of subjective involvement?

**DOI:** 10.3389/fnhum.2024.1359841

**Published:** 2024-03-12

**Authors:** Tom Froese, Chen Lam Loh, Finda Putri

**Affiliations:** Embodied Cognitive Science Unit, Okinawa Institute of Science and Technology Graduate University (OIST), Okinawa, Japan

**Keywords:** EEG, hyperscanning, joint action, neural synchrony, social interaction, neural entropy

## Abstract

Hyperscanning approaches to human neuroscience aim to uncover the neural mechanisms of social interaction. They have been largely guided by the expectation that increased levels of engagement between two persons will be supported by higher levels of inter-brain synchrony (IBS). A common approach to measuring IBS is phase synchrony in the context of EEG hyperscanning. Yet the growing number of experimental findings does not yield a straightforward interpretation, which has prompted critical reflections about the field’s theoretical and methodological principles. In this perspective piece, we make a conceptual contribution to this debate by considering the role of a possibly overlooked effect of inter-brain desynchronization (IBD), as for example measured by decreased phase synchrony. A principled reason to expect this role comes from the recent proposal of irruption theory, which operationalizes the efficacy of a person’s subjective involvement in behavior generation in terms of increased neural entropy. Accordingly, IBD is predicted to increase with one or more participant’s socially motivated subjective involvement in interaction, because of the associated increase in their neural entropy. Additionally, the relative prominence of IBD compared to IBS is expected to vary in time, as well as across frequency bands, depending on the extent that subjective involvement is elicited by the task and/or desired by the person. If irruption theory is on the right track, it could thereby help to explain the notable variability of IBS in social interaction in terms of a countertendency from another factor: IBD due to subjective involvement.

## 1 Introduction

Humans have a remarkable capacity for forming social collectives. The behavior of the smallest unit of collectives—dyadic interaction of a pair—has been variously studied in the cognitive sciences, for example as joint action, collective intentionality, we-mode, mutual incorporation, and embodied intersubjectivity. A novel theoretical framework that is emerging from these lines of research is that how we interact with others is irreducibly other-involving (Dumas et al., 2014), especially when there is emotional engagement (Schilbach et al., 2013). This kind of second-person framework fits naturally with the fact that some of the most meaningful experiences in our lives are moments that we share with others. The possibility that two or more people are subjectively involved in one shared experience has been referred to as “genuine intersubjectivity” to distinguish it from the traditional premise of a strict methodological individualism about social experience (Froese, 2018).

The neural mechanisms supporting social interaction are an active topic of research, especially by means of the application of inter-brain synchrony (IBS) and functional network measures to various hyperscanning approaches in the field of human neuroscience (Czseszumski et al., 2020). For the case of investigating the neural basis of naturalistic social interaction at fine-grained temporal resolution, EEG hyperscanning is the established method. Drawing inspiration from the hypothesized role of neural synchrony as a mechanism for large-scale integration of *intra-*brain activity (Varela et al., 2001), a guiding hypothesis of current efforts is to elucidate the role of IBS for large-scale integration of *inter-*brain activity (Müller, 2022). Various frequency ranges are expected to play a role in IBS, with faster ranges, especially gamma, suspected to be especially important for the integration of the neural basis of conscious experience across individuals (Valencia and Froese, 2020).

From a theoretical perspective, despite early recognition of a diversity of conceptualizations of social interaction (Pfeiffer et al., 2013), the field of second-person neuroscience has been predominantly driven by a search for increased IBS. Continued investigation of IBS is justified, as there is growing evidence of the causal role played by IBS in social interaction (e.g., Szymanski et al., 2017; Pan et al., 2021), yet the results of an expanding list of tasks do not always lend themselves to be interpreted in unidirectional increased in IBS (Hamilton, 2021; Holroyd, 2022). Moreover, the field does not yet have an explicit hypothesis to revise and broaden its experimental expectations, which hampers its capacity to see in which direction the rapidly accumulating data is pointing. If there are other factors at play other than IBS, then what are they and what is their role?

Accordingly, there is an opportunity, perhaps even a necessity, to complement the current largely data- and method-driven efforts in the field with a more explicit articulation of a theoretical framework of social interaction that can guide the process of scientific discovery. Steps in this direction had already been taken a decade ago by drawing on insights from embodied and enactive theory (e.g., Di Paolo and De Jaegher, 2012; Schilbach et al., 2013). But since then it has admittedly remained challenging to cross the gap from theory to experiment in the form of novel interpretations and testable predictions (Lehmann et al., 2023).

In this short perspective piece, we sketch the beginnings of a more differentiated theoretical framework for second-person neuroscience. Our proposal is based on the recent proposal of irruption theory (Froese, 2023), which takes inspiration from the neurophenomenology of embodied action (for a recent review, see Froese and Sykes, 2023). In a nutshell, our perspective amounts to the following novel claim: *a person’s subjective involvement in social interaction impacts their brain in the form of increased neural entropy, which will lead to inter-brain desynchronization (IBD)*. Accordingly, even if social interaction is generally associated with a tendency for increased IBS, presumably as a basis for interpersonal integration, any concomitant increase in subjective involvement is expected to also increase IBD, and hence to reduce the overall effect of IBS.

## 2 Irruption theory, neural entropy, and inter-brain synchrony

Irruption theory starts by taking seriously the insights derived by both our *first-person perspective* of lived experience, namely that we are agents who act in accordance with our motivations, and the *third-person perspective* of scientific observation, namely that we are complex physical systems that operate spontaneously in accordance with material constraints (Froese, 2023). The apparent tension between these two perspectives is resolved by highlighting that neither phenomenology, nor the physical sciences, can provide a complete description of human behavior on its own; they are limited in scope by the opaqueness of the lived body and the uncertainty of the living body, respectively. These foundational assumptions of irruption theory are captured by a set of three axioms:

1. Motivational efficacy: An agent’s motivations, *as such*, make a difference to the material basis of the agent’s behavior.
2. Incomplete materiality: It is impossible to measure how motivations, *as such*, make a difference to the material basis of behavior.
3. Underdetermined materiality: An agent’s behavior is underdetermined by its material basis.

Each of these axioms has been defended by different traditions in the literature, and taken together they come close to an embodied interpretation of the libertarian philosophical tradition on free will (Fuchs, 2021). The novel contribution of irruption theory is to go one step further, and to derive a novel working hypothesis from the integration of axioms 1 and 2: *The more an agent’s embodied activity is motivated, the less that activity is determined by its material basis*. In other words, this theory provides a meaningful interpretation of unexplainable variability in neural dynamics: a specific portion of that variability—what is referred to as an *irruption*—is a logical consequence of accepting both (1) that there is irreducible motivational efficacy, and (2) that any such motivational efficacy is unintelligible *as such*, that is, as motivational in nature, within the domain of material constraints.

A key challenge for this theory is to explain how an irruption could make an effective difference to the agent’s behavior, given that its immediate impact on the body amounts to a disordering rather than organizing factor. For this purpose, three theses are proposed:

1. Irruption Thesis: The living body is organized as an *incomplete system* such that it is open to involvement of motivations via increased material underdetermination.
2. Scalability Thesis: The living body is organized as a *poised system* such that it amplifies microscopic irruptions to macroscopic fluctuations that can impact behavior.
3. Attunement Thesis: The living body is organized as an *attuned system* such that it responds to scaled up irruptions in a context-sensitive and adaptive manner.

In this regard, irruption theory is consistent with a host of proposals that highlight how our bodies are organized as self-producing, thermodynamically open systems, situated dynamically at the edge of chaos, with a meta-stable grip on the world (for more detailed comparisons, see, e.g., Froese and Karelin, 2023; Froese et al., 2023). Its specific contribution is an alternative conceptualization of how to cash out the role of subjectively guided mental activity non-reductively: not in terms of the popular appeal to top-down constraints (e.g., Kelso, 1995; Freeman, 1999; Juarrero, 1999; Deacon, 2012), but rather as the destabilization of such spontaneously emergent constraints by irruptions. This is not the place to review the neuroscientific evidence in favor irruption theory, but it is consistent with a growing body of evidence that neural entropy tracks cognition and consciousness (e.g., Carhart-Harris et al., 2014; Lynn et al., 2021; Deco et al., 2022). More specifically, increased neural fluctuations have independently been proposed as the marker for volitional action in the context of the neuroscience of free will (Schurger et al., 2021), and the concept of irruption enables us to make sense of why volition manifests in precisely this way.

In the case of solitary situations, all cognitive tasks involve increased neural entropy production when compared to the resting state (Lynn et al., 2021), and the increase is particularly notable for tasks involving motor coordination, reward and decision making, and higher-order relational perceptual processing. Solitary tasks involving stimuli that represent social situations, e.g., auditory sentence and animation presentations, are associated with medium levels of entropy production. Even leaving aside the interpretative framework of irruption theory, it is reasonable to assume that the neural dynamics associated with real-time embodied social interaction would involve a combination of these task elements, and hence would exhibit elevated neural entropy. What does this kind of entropic disordering of brain dynamics mean for interbrain synchrony (IBS)?

As a first step, let us consider the origins of a popular method for calculating IBS, namely in terms of phase synchrony measured by phase-locking value (PLV). This measure was developed in Varela’s group (Varela et al., 2001) and first published by Lachaux et al. (1999). It was applied by Rodriguez et al. (1999) to the neural basis of the perceptual experience, where they found that a meaningful visual stimulus is associated with higher PLV and power in the gamma frequency spectrum.

However, Rodriguez et al. (1999) also observed desynchronization alternating with moments of increased synchrony. Although their focus was on synchrony as a candidate mechanism for perceptual binding or “neuronal glue,” the extent of desynchronization seems longer and larger: averaging over the trial would have presumably showed decreased PLV values for meaningful compared to non-meaningful stimuli. Prefiguring irruption theory, they interpreted this prominent desynchronization as a “process of active uncoupling of the underlying neural ensembles that is necessary to proceed from one cognitive state to another.” Indeed, to the extent that the formation of the next cognitive state is a self-organizing process based on the emergence of a cell assembly, this *neural synchronization* is, from the perspective of the person, a *passive* happening that is organizing behavior outside of their awareness and control. Hence, Varela’s (1995) intuition was that it is rather the moment of *neural desynchronization* that is indicative of the person’s *active* doing, that is, of their subjective involvement. Again, irruption theory enables us to explain why the efficacy of their subjective involvement would manifest in precisely this way.

There are important lessons for EEG hyperscanning that can be derived from irruption theory and the original analysis by Varela’s group of PLV during meaningful perception. We propose two novel hypotheses for future experimental testing:

- Across time, inter-brain synchrony (IBS) is expected to vary in accordance with the level of subjective involvement in the social interaction, and
- Averaged over time, increased subjective involvement in social interaction, due to more self- and/or other-related motivations, is expected to result in comparatively lower IBS.

We will now briefly explore these hypotheses in more detail.

## 3 Degrees of social engagement

**Figure 1:**
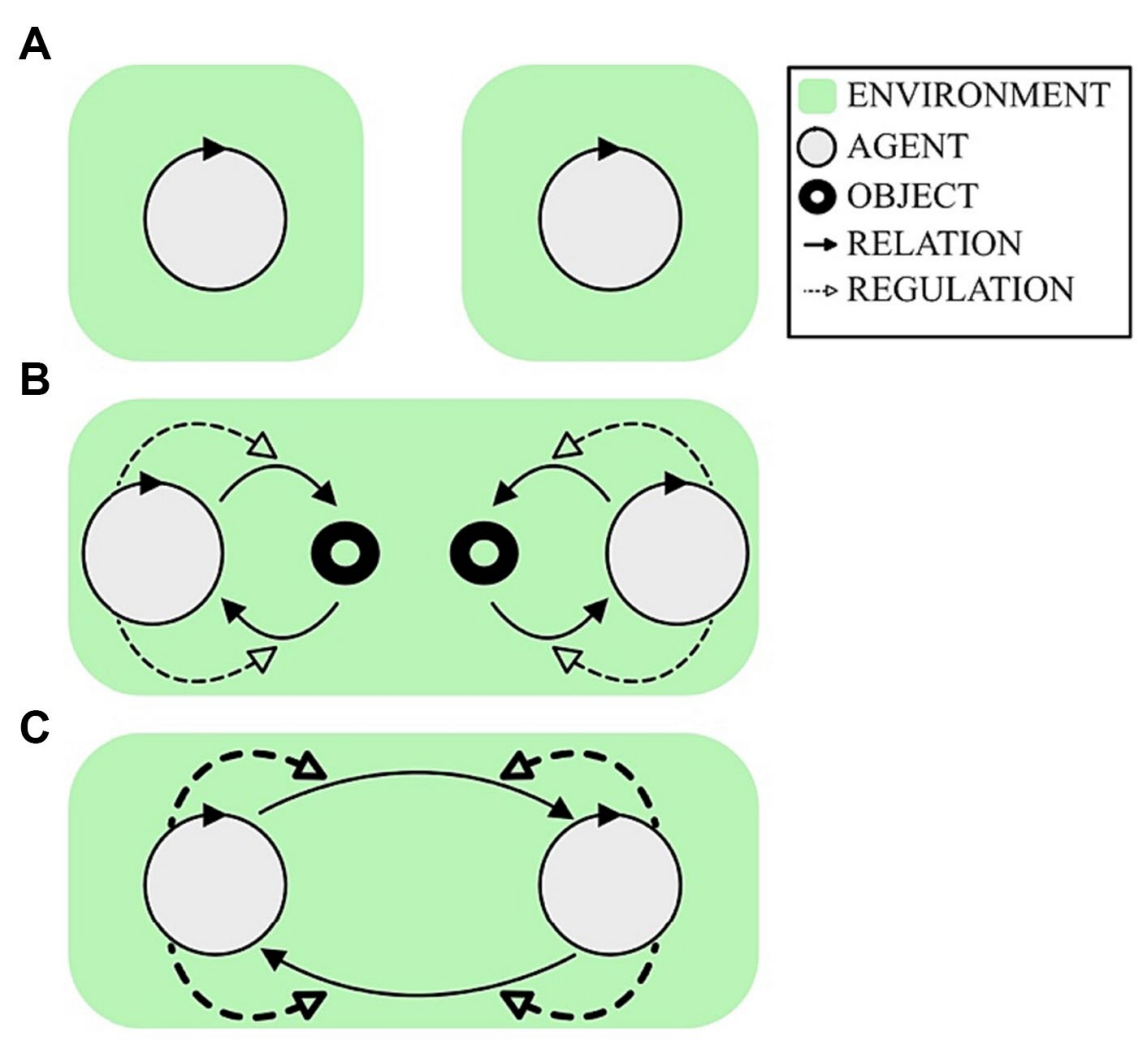
Three typical hyperscanning situations. Green represents the environment for each participant. A circular arrow represents a participant as an autonomous agent, following the autopoietic enactive tradition (Di Paolo et al., 2017). The outgoing and incoming black arrows represent the sensorimotor loop of how the agent is affecting and being affected by the environment, respectively. The dashed arrows indicate the agent’s active regulation of that sensorimotor loop to engage with the environment. **(A)** Simultaneous recording of resting state condition. **(B)** Two agents can engage in a task involving others, but in such a way that independent behavior regulation is largely sufficient to succeed, such as in many joint action tasks. **(C)** For some tasks, agents co-regulate how they affect each other in an interdependent manner, such as in practices of joint improvisation. How should we expect inter-brain synchrony (IBS) to vary across these conditions?

The EEG hyperscanning literature has largely ignored the role of individual brain variability when considering IBS in the context of different degrees of social engagement. The general expectation is of a positive association between the extent of inter-personal integration and the extent of IBS, for example based on the self-organization of hyper-brain cell assemblies (Müller, 2022). Consider the following three typical hyperscanning scenarios, as illustrated in Figure 1:

- An EEG recording of two independent people in resting state at the same time, as sometimes performed to establish a baseline condition;
- An EEG recording when the pair independently perform complex behaviors with respect to a shared task space, as typically studied in the context of joint action;
- An EEG recording when the pair engage in an interdependent reciprocally regulated interaction, as typically studied in the context of collective improvisation.

As an illustrative example of scenario B, consider the performance of pre-trained, pre-scripted behavioral sequences by duetting musicians (e.g., Gugnowska et al., 2022). A minimal version of scenario C could then involve the introduction of an unexpected perturbation to which the musicians have to interactively adapt (Lender et al., 2023). Most researchers engaged in hyperscanning would presumably expect that average IBS would come out as follows: (C) > (B) > (A). But is it so?

Regarding the resting state condition, we may still expect some degree of IBS simply because there is a nonzero probability that neural activity will exhibit similarity (Moreau et al., 2022). Moreover, if the resting state of the pair of participants was recorded synchronously, then the neural activity of both individuals is implicitly temporally aligned, which could elevate IBS. For example, they have been culturally integrated into the same universal clock system, and they began the resting state condition at the same instant. It is standard practice to consider such externally induced IBS as spurious and to remove it from further analysis (Holroyd, 2022). However, research employing multi-brain stimulation suggests that such externally induced IBS may still be efficacious for facilitating social coordination (Novembre and Iannetti, 2021), in which case there actually may be no such thing as spurious IBS. Future work may need to reconsider what counts as baseline, truly inefficacious IBS.

In the case of two participants independently working on a shared task, we may expect average IBS to be increased by the fact that neural activity of both participants is now externally synchronized not only by background temporal alignment, but also by other spatial and environmental factors favoring situational integration, such as shared stimuli (Hamilton, 2021). In addition, there is an expectation that working toward a shared goal will bring about, and in turn be further facilitated by, increased IBS (Szymanski et al., 2017), as also confirmed in the duetting musicians (Gugnowska et al., 2022).

However, compared to the resting state, each of the person’s perspective on the shared environment is unique and distinctive, and they will need to regulate their specific actions accordingly. Moreover, even if the task were to involve synchronized identical gestures, such as during action imitation, there is an added element of subjective involvement if imitation occurs spontaneously (Dumas et al., 2012). From the perspective of irruption theory, this individually and socially motivated increase in subjective involvement would manifest as increased neural entropy at the individual brain level, which would presumably show up as lower power at the intra-brain level and lower synchrony at the inter-brain level. For example, consider a musician’s brief disassociation from duetting, e.g., by making an unexpected change independently of the other’s behavior. This constitutes an individual perturbation to their joint performance followed by coordinated compensatory adjustments. As we would expect, the immediate decrease in socially motivated subjective involvement is characterized by relatively higher IBS, while the subsequent socially motivated re-alignment stands out because of its relatively higher IBD (Lender et al., 2023).

**Figure 2:**
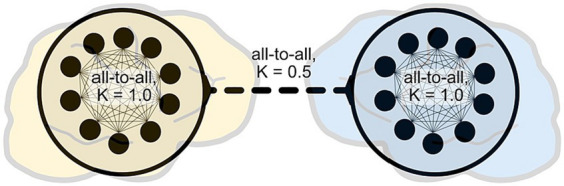
A highly simplified model of EEG hyperscanning. Following previous modeling work, we employed coupled Kuramoto oscillators to model the periodic activity of neurons or neuronal cell assemblies. This model is intended as a basic conceptual proof-of-concept to illustrate the possible consequences of increased intra-brain complexity on inter-brain synchrony; it does not make claims of biological realism. The code for this model has been made available in an online repository (https://gitlab.com/oist-ecsu/ibdesync).

As a basic proof of concept, we tested these ideas with a highly simplified model of the general EEG hyperscanning situation, following previous modeling work that used Kuramoto oscillators for this purpose (e.g., Dumas et al., 2012; Heggli et al., 2019; Loh and Froese, 2021; Moreau et al., 2022). Each artificial “brain” consisted of 10 oscillators with intra- and inter-brain coupling strengths set to 1 and 0.5, respectively. Apart from the lower coupling strength of inter-brain coupling, we did not impose any further “behavioral bottleneck” (Kingsbury and Hong, 2020) to avoid biasing the model against IBS (Figure 2).

The proposed situation of an irruption-based increase in neural entropy, as a manifestation of subjective involvement in social interaction, was modeled in terms of an availability of a broader range of neural frequencies. Note that this increase in neural state space is a conservative choice of modeling irruptions, because their disordering impact may be better captured by an increase in aperiodic neural activity. We compared two conditions C1 and C2: in C1 the natural frequency range of was taken from a uniform distribution of [39, 41], whereas in C2 the range was wider [30, 50]. Each condition was simulated 1,000 times; IBS was calculated using the circular correlation coefficient (CCorr), as advocated by Burgess (2013), and all correlation coefficients were averaged per condition. We found that in C2, compared to C1, the average intra-brain CCorr decreased by more than half, and as expected the inter-brain CCorr also decreased, namely by nearly an order of magnitude (C1: 0.32. C2: 0.045). To be fair, this is only the most minimal proof of concept. Future work could test our predictions more systematically by implementing models with biological realism, and with targeted analysis of experimental data.

## 4 Discussion

Social neuroscience approaches have been predicting that increased social engagement and interpersonal integration, such as shared goals in joint action (Zamm et al., 2023), is generally associated with increased IBS across brains and bodies. We have complemented this standard prediction with the working hypothesis of irruption theory, namely that increased subjective involvement will manifest as increased neural entropy (Froese, 2023), and hence will act as a countertendency of desynchronization in the intra- and inter-brain levels of analysis.

If our theoretical perspective is on the right track, we may wonder why there is not yet significant evidence for the importance of IBD in social interaction, especially when compared to well-known findings of IBS. On the one hand, it is possible that the effect of IBD is equivalent to IBS, thereby leading to null results after averaging, or perhaps the effect of IBD is comparatively smaller when compared to IBS. However, given the field’s strong bias toward finding IBS as the main marker of social interaction, concerns have already been raised that this narrow focus may fail to capture other relevant features (Hamilton, 2021), and that there may have been a factor of IBS “confirmation bias” (Holroyd, 2022). Possibly, null results or contrary findings of significantly increased IBD that did not fit theoretical expectations perhaps did not reach publication stage. It is our hope that this perspective piece helps to broaden the range of hyperscanning findings that can be predicted and interpreted.

Could IBD have a positive role to play in itself? We suggest that IBD is accentuated when the normative conditions guiding behavior are not limited to one person, but are distributed over two or more individuals. Prime examples are turn-taking and giving-taking kinds of social interaction, in which success of one’s behavior is dependent on the other’s complementary behavior (De Jaegher and Di Paolo, 2008). In these situations, irruption theory predicts that the increased subjective involvement in social interaction will have the paradoxical effect of impeding the neural basis of social integration. This injection of IBD in the context of increased IBS may seem counterproductive at first, but it could facilitate the kinds of flexible cognitive-behavioral transitions that characterize normal social coordination (Di Paolo and De Jaegher, 2012). And, conversely, a neural mechanism for the prevention of excessive social integration could be essential for the maintenance of mental health, and may be impaired in some conditions (Galbusera et al., 2019; Froese and Krueger, 2021).

Variability of IBS over time has been known about for some time (Dumas et al., 2010), but it has only recently received renewed attention in the hyperscanning literature (e.g., Li et al., 2021; Haresign et al., 2022; Wikström et al., 2022). Future work could aim to systematically quantify IBS variability as the expected multi-brain signature of a healthy, spontaneously motivated social interaction. We suggest that IBS variability should be understood as the natural expression of the flexible balancing required to coordinate two competing dynamical tendencies, namely IBS and IBD, which are associated with interpersonal integration and subjective involvement, respectively.

## Data availability statement

The original contributions presented in the study are included in the article/supplementary material, further inquiries can be directed to the corresponding author.

## Author contributions

TF: Conceptualization, Investigation, Supervision, Writing – original draft, Writing – review & editing, Funding acquisition, Project administration. CL: Conceptualization, Formal analysis, Investigation, Visualization, Writing – review & editing. FP: Investigation, Writing – review & editing, Conceptualization.
